# Baseline PET/CT metabolic parameters in the double-expressor subtype of diffuse large B-cell lymphoma: development of a clinical–radiologic–pathologic predictive model

**DOI:** 10.3389/fonc.2025.1660516

**Published:** 2025-11-28

**Authors:** Jingjing Liang, Guoxiu Lu, Qi Peng, Jigang Wang, Guoxu Zhang

**Affiliations:** 1 Department of Nuclear Medicine, General Hospital of Northern Theatre Command, Shenyang, China; 2 Training Base for Graduate, General Hospital of Northern Theater Command, China Medical University, Shenyang, China; 3 Department of Hematology, General Hospital of Northern Theatre Command, Shenyang, China

**Keywords:** diffuse large B-cell lymphoma, double-expression lymphoma, ^18^F-FDG PET/CT, c-Myc, Bcl-2, predictive model

## Abstract

**Background:**

Double-expressor lymphoma (DEL) is an aggressive diffuse large B-cell lymphoma (DLBCL) subtype (20%–30% of cases) exhibiting resistance to standard immunochemotherapy regimens [R-CHOP (rituximab, cyclophosphamide, doxorubicin, vincristine, and prednisone)]. Multiple clinical studies have demonstrated that the combined use of new drugs such as Chidamide can significantly improve outcomes in DEL, underscoring the need for early identification of high-risk patients to guide therapy. In this context, baseline ^18^F-FDG positron emission tomography/computed tomography (PET/CT) metabolic parameters are poised to become a pivotal tool for optimizing risk stratification in DEL, owing to their unique capability to non-invasively quantify tumor metabolic heterogeneity.

**Methods:**

We retrospectively analyzed clinical and baseline ^18^F-FDG PET/CT imaging data from treatment-naive patients with DLBCL at the Northern Theater Command General Hospital from January 2020 to February 2025. Patients were classified into a DEL group and a non-DEL group. PET/CT parameters were correlated with clinical–pathologic features using Spearman analysis. Optimal thresholds for maximum standardized uptake value (SUVmax), total metabolic tumor volume (TMTV), and total lesion glycolysis (TLG) were determined by receiver operating characteristic (ROC) analysis. Univariate and multivariate analyses were performed to identify independent predictors, followed by the development and validation of a combined prediction model using ROC analysis, calibration curves, and decision curve analysis (DCA).

**Results:**

A total of 128 patients (71 men and 57 women, median age, 60 years, range, 16 to 87 years) were included in the non-DEL group (*n* = 82) and DEL group (*n* = 46). Spearman analysis revealed that PET/CT parameters significantly correlated with the International Prognostic Index (IPI), Ann Arbor stage, B symptoms, β2-MG, LDH, and non-GCB (*r* = 0.20–0.68, *p* < 0.05), but not Ki-67 (*p* > 0.05). TMTV demonstrated optimal DEL prediction (threshold = 510.22 cm³, AUC = 0.81, 95% CI, 0.73–0.88 *p* = 0.038). TMTV (>510.22 cm³, OR = 8.79, 95% CI, 3.20–24.11, *p* < 0.001), IPI (OR = 3.82, 95% CI, 1.44–10.11, *p* = 0.007), and Ki-67 (OR = 1.07, 95% CI, 1.03–1.11, *p* = 0.001) were identified as independent DEL predictors. The TMTV+IPI+Ki-67 combined model (AUC = 0.867, *p* < 0.05) showed significantly higher discriminative performance compared to dual-parameter models (TMTV+IPI, AUC = 0.798; TMTV+Ki-67, AUC = 0.844; IPI+Ki-67, AUC = 0.797, all *p* < 0.05). This superiority was further confirmed through calibration curves and DCA, indicating its reliable predictive accuracy and clinical utility.

**Conclusions:**

TMTV, IPI, and Ki-67 are robust independent predictors of DEL. The integrated clinical–imaging–pathological prediction model constructed from these three parameters synergistically combines multi-dimensional information, significantly enhancing early DEL identification capability and facilitating the implementation of risk-adapted therapeutic strategies.

## Introduction

Diffuse large B-cell lymphoma (DLBCL), the most common subtype of non-Hodgkin lymphoma (NHL), constitutes approximately 40% of adult NHL cases (1). Its marked clinical heterogeneity poses a major challenge for precision therapy. Molecular subtyping advances have progressively elucidated the substantial heterogeneity within DLBCL. The co-overexpression of c-Myc and Bcl-2 proteins detected by immunohistochemistry (IHC) defines double-expressor lymphoma (DEL), representing 20%–30% of newly diagnosed DLBCL cases (2, 3). DEL typically exhibits resistance to standard R-CHOP immunochemotherapy (rituximab, cyclophosphamide, doxorubicin, vincristine, and prednisone) and demonstrates strong associations with adverse prognostic features, including elevated Ki-67, increased lactate dehydrogenase (LDH) levels, and more advanced Ann Arbor stage (4–6). While novel agents (e.g., histone deacetylase inhibitors such as Chidamide) combined with R-CHOP may improve DEL outcomes (7–11), current diagnostic identification relies solely on pathological criteria—a method that is time-consuming and carries inherent risks of specimen damage during handling. Consequently, developing early-detection strategies for DEL to guide treatment intensification remains a critical unmet clinical need.

^18^F-FDG positron emission tomography/computed tomography (PET/CT) metabolic parameters objectively quantify tumor proliferative activity and metabolic burden, effectively evaluating disease status. This modality corrects staging in 15%–20% of patients and modifies therapeutic strategies in nearly 8% of cases, and is strongly recommended for routine use in initial staging and treatment response evaluation of DLBCL (12). Maximum standardized uptake value (SUVmax) captures peak metabolic activity within lesions, reflecting tumor glycolytic intensity. Metabolic tumor volume (MTV), as a measurable volumetric parameter, objectively quantifies the whole-body metabolic tumor burden. Total lesion glycolysis (TLG) integrates both metabolic volume and glycolytic intensity. Collectively, these parameters provide a comprehensive assessment of tumor metabolic heterogeneity (14) and demonstrate significant positive correlation with lymphoma aggressiveness, holding independent prognostic value for DLBCL risk stratification (15–18). Notably, Dong et al. (19) demonstrated that SUVmax significantly correlates with c-Myc/Bcl-2 protein expression and directly impacts DLBCL treatment efficacy. Zhao et al. (13) established a total metabolic tumor volume (TMTV)-based grading system predictive of outcomes in DEL. These findings collectively suggest that PET/CT metabolic parameters may serve as noninvasive imaging biomarkers for identifying DEL, with substantial translational significance for personalized therapy. However, systematic research on PET/CT for DEL diagnosis, prognostication, and treatment response prediction remains insufficient.

This study aims to incorporate baseline PET/CT metabolic parameters, clinical characteristics, and pathological biomarkers to comprehensively investigate the association between metabolic imaging features and DEL molecular phenotypes. We subsequently construct a multimodal early-detection model for DEL, seeking to overcome the limitations of conventional risk stratification and provide a precise clinical tool for early intervention and individualized therapy in high-risk patients.

## Materials and methods

### Population

We retrospectively analyzed clinical characteristics and baseline PET/CT metabolic parameters in 128 treatment-naive patients with DLBCL (71 men and 57 women, median age, 60 years, range, 16 to 87 years) evaluated at our institution from January 2020 through February 2025. Patients were stratified into a non-DEL (*n* = 82) group and a DEL (*n* = 46) group based on immunohistochemical criteria (c-Myc ≥ 40% and Bcl-2 ≥ 50%) (2).

The inclusion criteria were as follows, (i) pathologically verified DLBCL with complete IHC; (ii) baseline ^18^F-FDG PET/CT images (≥1 lesion with SUVmax > 2.5); and (iii) availability of complete clinical data. The following were the exclusion criteria, (i) central nervous system lymphoma (CNSL) (20) or secondary malignancies; (ii) history of prior antitumor therapy (except for diagnostic biopsies or excisional lymph node biopsies); and (iii) incomplete clinical data or poor-quality PET/CT images.

**Figure 1:**
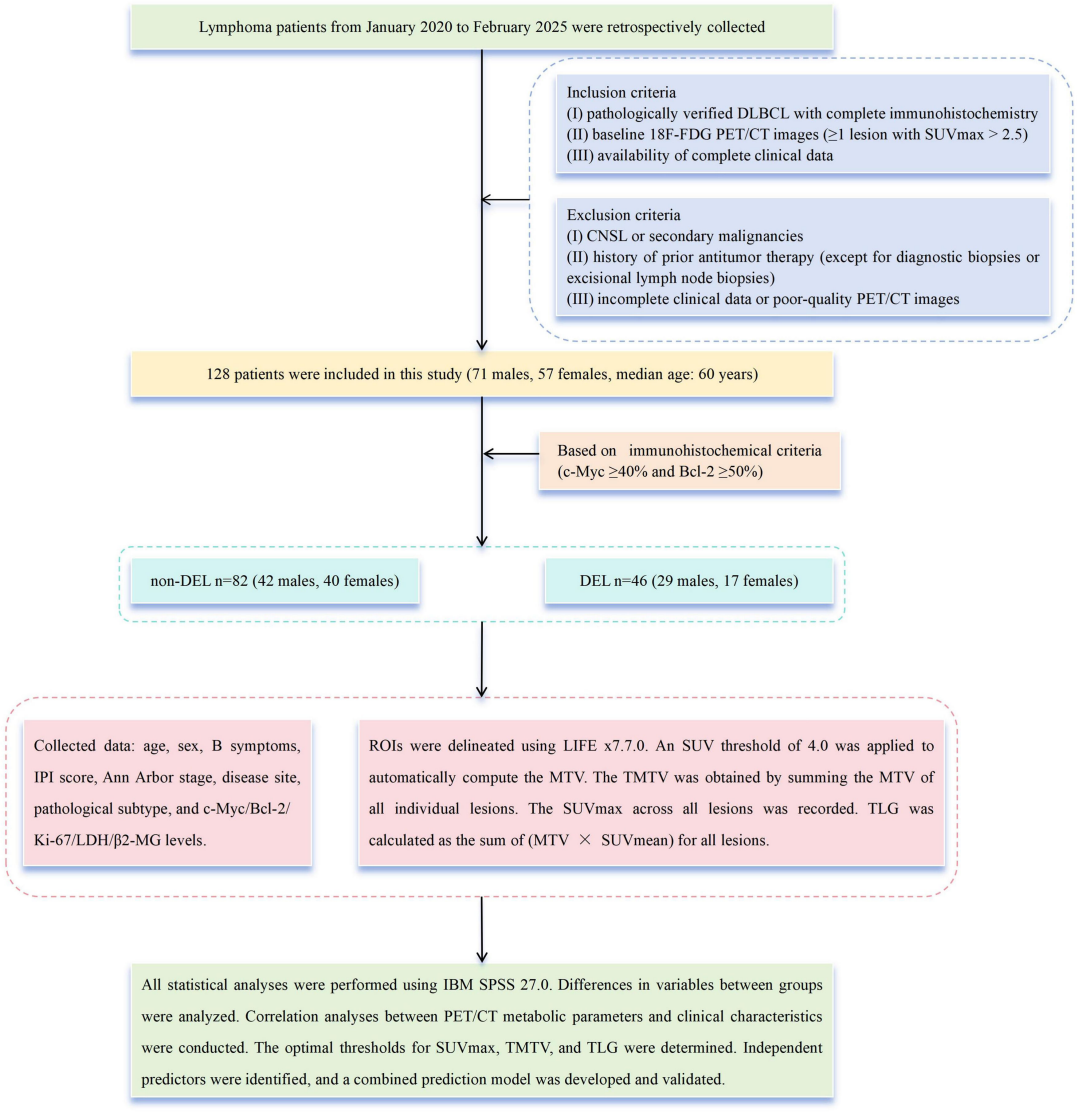
DLBCL patients enrollment flowchart.

Collected data from medical records included the following, age, sex, B symptoms, International Prognostic Index (IPI) score (0–2/3–5), Ann Arbor stage (I–II/III–IV), disease site (nodal/extranodal), pathological subtype (GCB/non-GCB), expression levels of c-Myc, Bcl-2, Ki-67, LDH, and β2-microglobulin (β2-MG) (**Figure 1**).

The study was conducted in accordance with the Declaration of Helsinki and approved by the Ethics Committee of Northern Theater Command General Hospital (Approval No. Y[2025]187). As a retrospective study, it was exempt from the requirement of obtaining informed consent.

### ^18^F-FDG PET/CT acquisition

All scans utilized the Discovery PET/CT system (GE Healthcare). Patients fasted ≥6 h (blood glucose < 11.1 mmol/L). According to body weight, 3.70–5.18 MBq/kg of ^18^F-FDG (produced by a GE Healthcare medical cyclotron and synthesized via an automatic synthesis module, with radiochemical purity >98%) was intravenously injected. After a 50- to 60-min quiet rest period, scanning commenced. Routine scans were conducted from the skull base to the mid-thigh level, with additional coverage extended to the feet when necessary. CT scanning parameters included the following, tube voltage, 120 kV; tube current, 110 mA; pitch, 1.0; rotation time, 0.5 s/rotation; and slice thickness, 3.27 mm. PET scanning employed 3D acquisition with six to eight bed positions at 1.5 min per bed position. CT data were used for automated attenuation correction of PET images.

### ^18^F-FDGPET/CT image analysis

**Figure 2:**
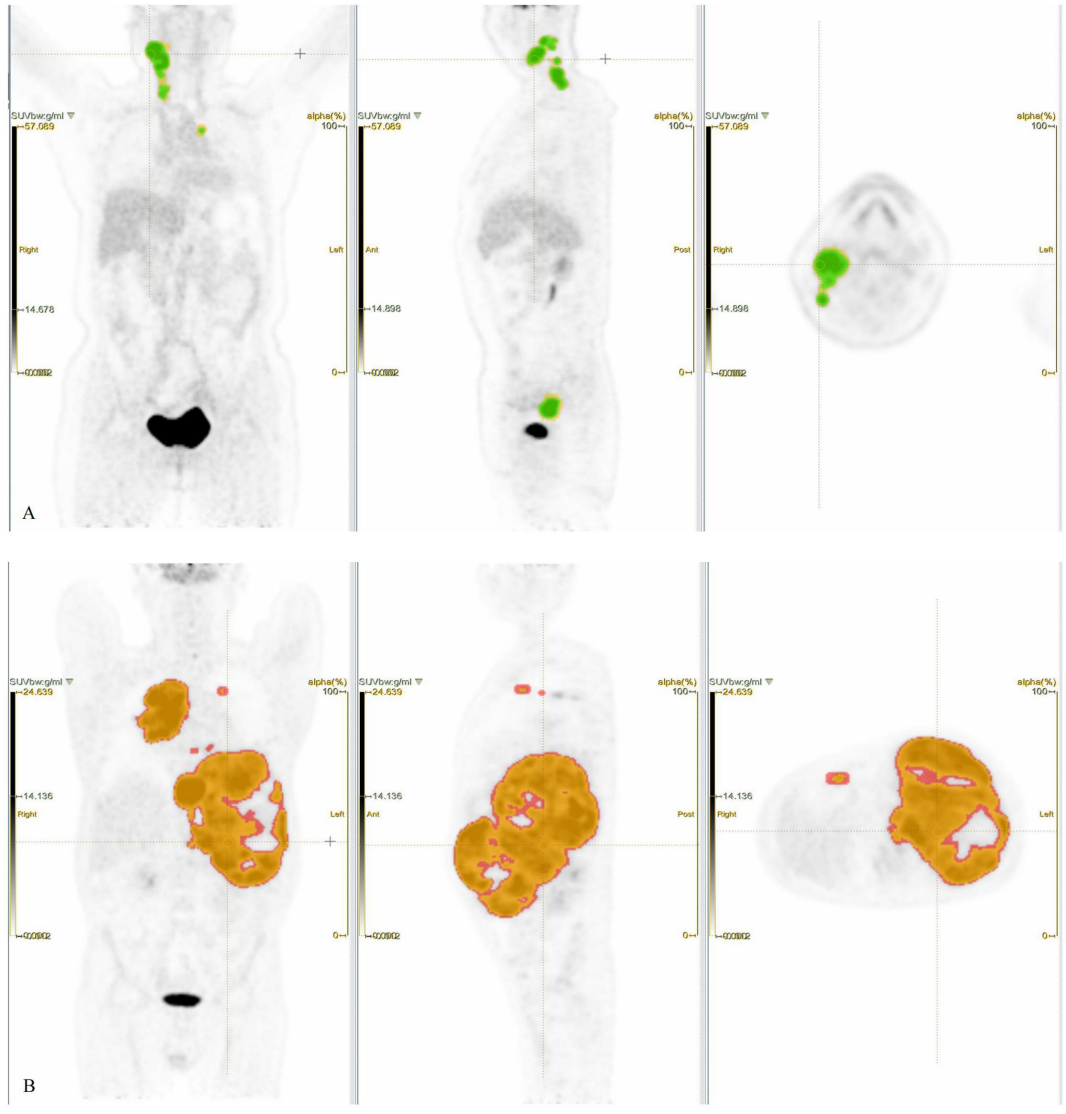
Patient 1, female, 59 years old, presenting with a painful, firm right cervical lymphadenopathy (4 × 4 cm) persisting for over 3 months, without fever, night sweats, or significant weight change. Laboratory findings, LDH 160 U/L, β2-MG 1.80 mg/L. Pathology, DLBCL. IHC, c-Myc 20%, Bcl-2 −, Ki-67 50%. Clinical diagnosis, DLBCL (non-GCB), stage IIA, IPI score 1 (low risk). **(A)** PET ROIs, where the yellow ROI denotes total tumor volume and the green overlapping ROI (SUV ≥ 4.0) was used to calculate TMTV and TLG. (Note, Not all lesion areas are displayed.) Patient 2, male, 53 years old. Intermittent abdominal pain for over 4 months without fever or night sweats, no significant weight change. Physical examination, Abdominal distension, spleen palpable 4–5 cm below the left costal margin along Schmid’s line I, 8–11 cm along Schmid’s line II, and +3 cm along Schmid’s line III. No other positive signs. Laboratory findings, LDH, 1,385 U/L, β2-MG, 6.23 mg/L. Pathology, DLBCL (DEL). IHC, c-Myc 50%, Bcl-2 90%, Ki-67 80%. Clinical diagnosis, DLBCL (non-GCB) Stage IV A, IPI score, 4 points (high risk). **(B)** PET ROIs, where the red ROI denotes total tumor volume and the orange overlapping ROI (SUV ≥ 4.0) was used to calculate TMTV and TLG. (Note, Not all lesion areas are displayed.).

Images of regions of interest (ROIs) were delineated using LIFEx7.7.0 (21), with careful exclusion of physiological high-uptake regions such as the brain, heart, and bladder to minimize impact on results. Initially, ROI was independently performed by a junior and a senior radiologist, and discrepancies (>15% volume difference) were resolved by a senior nuclear medicine specialist. Secondly, SUV = 4.0 was applied to automatically compute the MTV (22–24). The TMTV was obtained by summing the MTV of all individual lesions. The SUVmax among all lesions was recorded. The TLG was the sum of MTV × SUVmean of all lesions (**Figure 2**).

### Statistical analysis

All statistical analyses used IBM SPSS 27.0. Categorical variables were presented as frequencies (%) and analyzed using *χ*^2^ tests. Continuous variables were reported as mean ± SD or median [interquartile range (IQR)], with comparisons performed via *t*-tests or Mann–Whitney *U* tests, respectively. PET/CT parameters were correlated with clinical–pathologic features using Spearman analysis. Optimal thresholds for SUVmax, TMTV, and TLG were determined by receiver operating characteristic (ROC) curves (Youden’s index). Univariate and multivariate analyses were conducted to identify independent predictors, followed by development and validation of a combined prediction model using ROC analysis, calibration curves, and decision curve analysis (DCA). A *p*-value < 0.05 was considered statistically significant.

## Results

### General features

**Table 1** summarizes the clinical characteristics of 128 enrolled patients. Compared to non-DEL patients, DEL patients exhibited significantly higher proportions of high-risk features, including B symptoms (58% vs. 32%, *p* = 0.005), advanced Ann Arbor stage (III–IV, 80% vs. 54%, *p* = 0.004), IPI ≥ 3 (76% vs. 32%, *p* < 0.001), and non-GCB subtype (67% vs. 48%, *p* = 0.042). Furthermore, DEL patients demonstrated significantly elevated median values across key metabolic and serologic biomarkers, LDH [360.50 U/L (IQR 238.25–529.75) vs. 219.50 U/L (IQR 170.75–316.75)], β2-MG [3.98 mg/L (IQR 2.84–5.03) vs. 2.84 mg/L (IQR 2.13–3.12)], Ki-67 [80.00% (IQR 70.00–86.25) vs. 80.00% (IQR 80.00–90.00)], and PET/CT parameters including SUVmax [29.07 (IQR 23.79–37.03) vs. 22.35 (IQR 16.47–34.04)], TMTV [7,761.36 cm^3^ (IQR 2,879.92–12,914.86) vs. 1,319.35 cm^3^ (IQR 310.01–4,290.68)], and TLG [763.54 g (IQR 311.48–1,221.14) vs. 151.74 g (IQR 41.09–487.90)]. The substantial IQRs underscore the marked disparities in these parameters.

**Table 1:** Comparative analysis of baseline characteristics and PET/CT metabolic parameters between non-DEL and DEL cohorts.

Characteristic	Variable	Number of cases		Statistic (*χ*²/*Z*)	*p*-value*
		Non-DEL group (*n* = 82)	DEL group (*n* = 46)		
Clinical features					
Categorical					
Sex				1.67	0.197
	Male	42 (51.2%)	29 (63.0%)		
	Female	40 (48.8%)	17 (37.0%)		
Age (years)				0.44	0.507
	≤60	46 (56.1%)	23 (50.0%)		
	>60	36 (43.9%)	23 (50.0%)		
Site				0.13	0.722
	Nodal	26 (31.7%)	16 (34.8%)		
	Extranodal	56 (68.3%)	30 (65.2%)		
B symptoms				8.02	0.005
	No	57 (67.1%)	19 (41.3%)		
	Yes	27 (32.9%)	27 (58.7%)		
Ann Arbor stage				8.36	0.004
	I-II	37 (45.1%)	9 (19.6%)		
	III-IV	45 (54.9%)	37 (80.4%)		
IPI				21.98	<0.001
	0–2	55 (67.1%)	11 (23.9%)		
	3–5	27 (32.9%)	35 (76.1%)		
Pathological type				4.13	0.042
	GCB	42 (51.2%)	15 (32.6%)		
	Non-GCB	40 (48.8%)	31 (67.4%)		
Continuous	Ki-67 (%)	80.00 (70.00, 86.25)	80.00 (80.00, 90.00)	−2.895	0.004
	β2-MG (mg/L)	3.98 (2.84, 5.03)	2.84 (2.13, 3.12)	−4.587	<0.001
	LDH (U/L)	360.50 (238.25, 529.75)	219.50 (170.75, 316.75)	−4.365	<0.001
Metabolic features					
Continuous	SUVmax	29.07 (23.79, 37.03)	22.35 (16.47, 34.04)	−3.469	0.001
	TMTV (cm^3^)	7,761.36 (2,879.92, 12,914.86)	1,319.35 (310.01, 4,290.68)	−5.656	<0.001
	TLG (g)	763.54 (311.48, 1,221.14)	151.74 (41.09, 487.90)	−5.771	<0.001
Categorical					
SUVmax				15.07	<0.001
	Low	45 (54.9%)	9 (19.6%)		
	High	37 (45.1%)	37 (80.4%)		
TMTV				29.75	<0.001
	Low	65 (79.3%)	14 (30.4%)		
	High	17 (20.7%)	32 (69.6%)		
TLG				23.27	<0.001
	Low	56 (68.3%)	11 (23.9%)		
	High	26 (31.7%)	35 (76.1%)		

Categorical variables are presented as frequencies (%) and analyzed using the *χ*² test (reported as *χ*² value). Continuous variables, owing to non-normal distribution, are presented as median (Q1, Q3) and analyzed using the Mann–Whitney *U* tests (reported with *Z*-score). Threshold definitions based on ROC optimal cutoffs, SUVmax, low ≤23.35, high >23.35; TMTV, low ≤510.22 cm^3^, high >510.22 cm^3^; TLG, low ≤2,994.85 g, high >2,994.85 g. Site, Lesion location (nodal/extranodal); Ann Arbor stage (I–II, early stage; III–IV, advanced stage); IPI, International Prognostic Index (0–2, low risk; 3–5, high risk); Pathological type (GCB, germinal center B-cell-like; non-GCB, non-germinal center B-cell-like subtypes); Ki-67, proliferation index; β2-MG, β2-microglobulin; LDH, lactate dehydrogenase; SUVmax, maximum standardized uptake value; TMTV, total metabolic tumor volume; TLG, total lesion glycolysis. *Significance level, *p* < 0.05.

### Correlations between PET/CT metabolic parameters and clinical features

**Figure 3:**
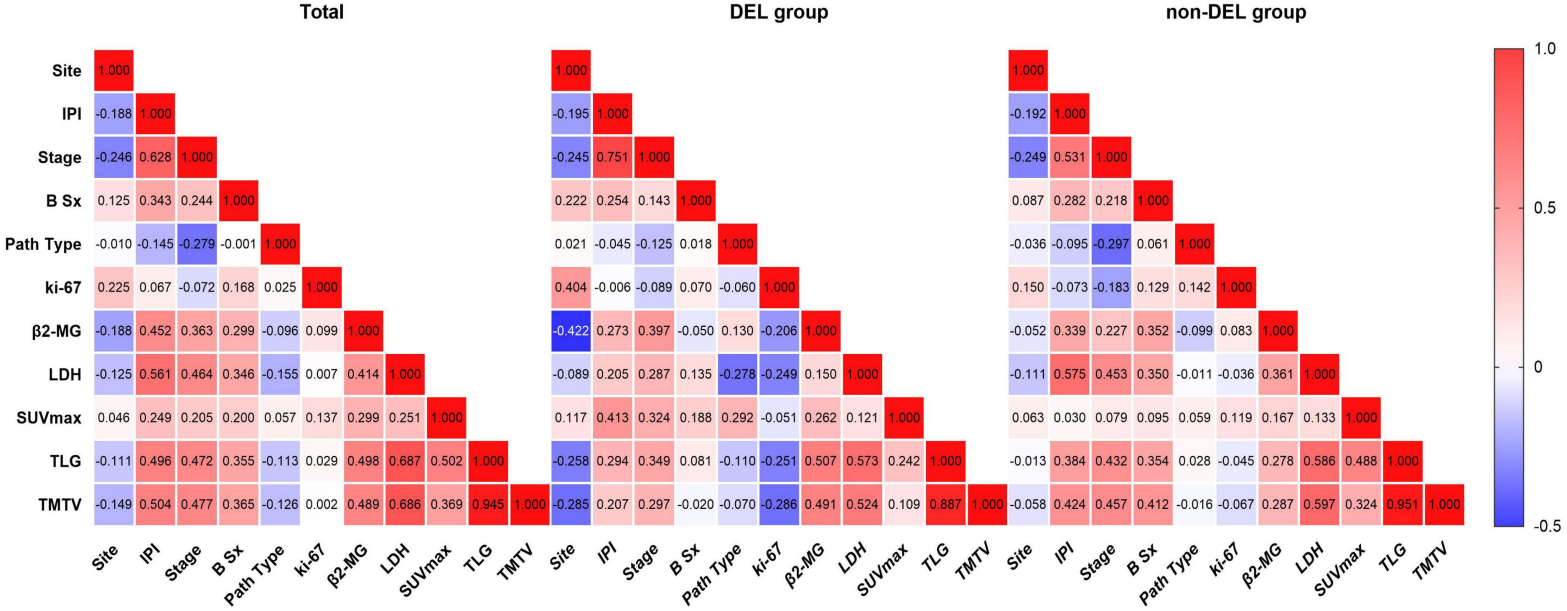
Correlations between PET/CT parameters and clinical features. From left to right are the overall cohort, the DEL group, and the non-DEL group, respectively. Site, Lesion location (nodal/extranodal); IPI, International Prognostic Index (0–2, low risk; 3–5, high risk); Stage, Ann Arbor stage (I–II, early stage; III–IV, advanced stage); B Sx, B symptoms; Path Type, pathological type (GCB, germinal center B-cell-like; non-GCB, non-germinal center B-cell-like subtypes); Ki-67, proliferation index; β2-MG, β2-microglobulin; LDH, lactate dehydrogenase; SUVmax, maximum standardized uptake value; TLG, total lesion glycolysis; TMTV, total metabolic tumor volume. Significance level, *p* < 0.05.

In the overall cohort (**Figure 3**), SUVmax/TMTV/TLG exhibited significant positive correlations with IPI (*r* = 0.249/0.496/0.504; *p* ≤ 0.005), Ann Arbor stage (*r* = 0.205/0.472/0.477; *p* ≤ 0.02), B symptoms (*r* = 0.200/0.355/0.365; *p* ≤ 0.024), β2-MG (*r* = 0.299/0.498/0.489; *p* ≤ 0.001), and LDH (*r* = 0.251/0.687/0.686; *p* ≤ 0.004), but not with pathological subtype (GCB/non-GCB), lesion site (nodal/extranodal), or Ki-67 (*p* > 0.05). In the DEL subgroup (**Figure 3**), SUVmax correlated with IPI (*r* = 0.413, *p* = 0.004), Ann Arbor stage (*r* = 0.324, *p* = 0.028), and pathological type (*r* = 0.292, *p* = 0.049). TMTV correlated with Ann Arbor stage (*r* = 0.297, *p* = 0.045), LDH (*r* = 0.524, *p* < 0.001), and β2-MG (*r* = 0.491, *p* = 0.001), while TLG correlated with IPI (*r* = 0.294, *p* = 0.048), Ann Arbor stage (*r* = 0.349, *p* = 0.018), β2-MG (*r* = 0.573, *p* < 0.001), and LDH (*r* = 0.573, *p* < 0.001). No parameter correlated with B symptoms or Ki-67 (*p* > 0.05). In the non-DEL subgroup (**Figure 3**), TMTV/TLG correlated with IPI (*r* = 0.424/0.384; *p* < 0.001), Ann Arbor stage (*r* = 0.457/0.432; *p* < 0.001), B symptoms (*r* = 0.412/0.354; *p* ≤ 0.001), β2-MG (*r* = 0.287/0.278; *p* ≤ 0.011), and LDH (*r* = 0.597/0.586; *p* < 0.001), but not with lesion site, pathological type, or Ki-67 (*p* > 0.05). SUVmax displayed no significant associations with any clinical features (*p* > 0.05). Notably, metabolic parameters consistently showed no correlation with Ki-67 across all subgroups (*p* > 0.05).

### ROC curve analysis

ROC analysis established optimal stratification thresholds for metabolic parameters, SUVmax at 23.35 (AUC = 0.685, *p* = 0.047), TMTV at 510.22 cm³ (AUC = 0.808, *p* = 0.038), and TLG at 2,994.85 g (AUC = 0.802, *p* = 0.039) (**Table 2**). Notably, both volumetric parameters (TMTV and TLG) demonstrated superior discriminatory capacity (AUC > 0.800) compared to SUVmax. Based on these thresholds, patients were stratified into low and high metabolic parameter groups, with significant differential distribution between non-DEL and DEL cohorts detailed in **Table 1**.

**Table 2:** ROC analysis for predicting DEL.

Variable	AUC (95% CI)	Sensitivity (%)	Specificity (%)	*p*-value*
B symptoms (no/yes)	0.629 (0.527–0.731)	58.7	67.1	0.016
Ann Arbor stage (I–II/III–IV)	0.628 (0.529–0.726)	80.4	45.1	0.017
IPI (0–2/3–5)	0.716 (0.623–0.809)	76.1	67.1	<0.001
Pathological type (GCB/non-GCB)	0.407 (0.305–0.509)	32.6	48.8	0.081
Ki-67 (%)	0.651 (0.555–0.747)	78.0	45.1	0.005
β2-MG (mg/L)	0.745 (0.653–0.836)	65.2	82.9	<0.001
LDH (U/L)	0.733 (0.646–0.820)	65.2	70.7	0.045
SUVmax	0.685 (0.594–0.777)	80.4	54.9	0.047
TMTV (cm^3^)	0.808 (0.734–0.882)	69.6	79.3	0.038
TLG (g)	0.802 (0.726–0.878)	76.1	68.3	0.039

Optimal thresholds for the metabolic parameters calculated by ROC analysis, SUVmax = 23.35, TMTV = 510.22 cm^3^, and TLG = 2,994.85 g. AUC, area under the curve; CI, confidence interval; Ann Arbor stage (I–II, early stage; III–IV, advanced stage); IPI, International Prognostic Index (0–2, low risk; 3–5, high risk); Pathological type (GCB, germinal center B-cell-like; non-GCB, non-germinal center B-cell-like subtypes); Ki-67, proliferation index; β2-MG, β2-microglobulin; LDH, lactate dehydrogenase; SUVmax, maximum standardized uptake value; TMTV, total metabolic tumor volume; TLG, total lesion glycolysis. *Significance level, *p* < 0.05.

### Univariate and multivariate analysis results

Univariate analysis identified IPI, Ann Arbor stage, B symptoms, LDH, β2-MG, Ki-67, SUVmax, TMTV, and TLG as predictors of DEL (all *p* < 0.05). Multivariate analysis identified TMTV (>510.22 cm³, OR = 8.79, 95% CI, 3.20–24.11, *p* < 0.001), IPI (OR = 3.82, 95% CI:1.44–10.11, *p* = 0.007), and Ki-67 (OR = 1.07, 95% CI, 1.03–1.11, *p* = 0.001) as independent predictors.

### Diagnostic prediction model development and evaluation

**Figure 4:**
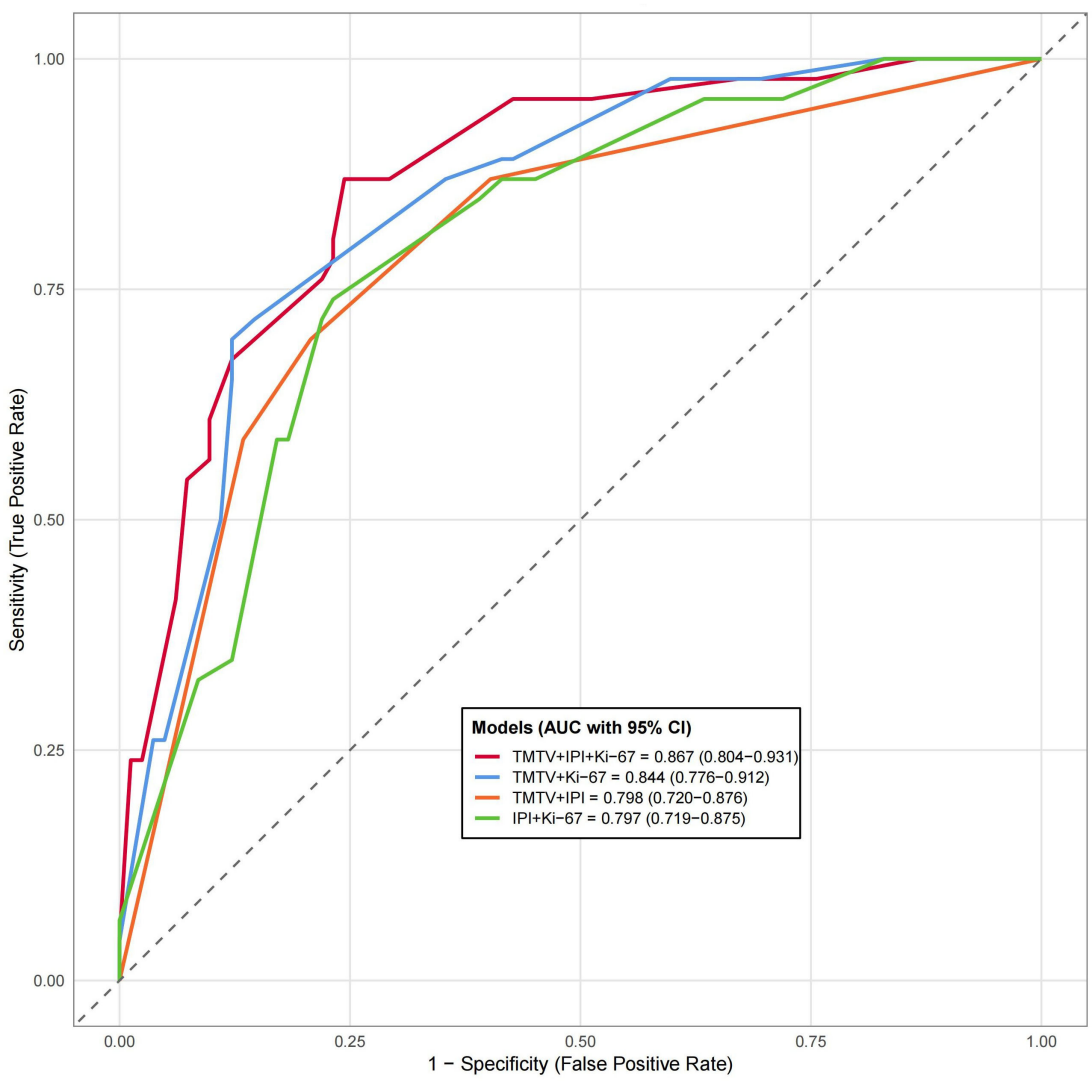
Compare the receiver working characteristic curve analysis of different models. AUC, area under the curve; CI, confidence interval; IPI, International Prognostic Index (0–2, low risk; 3–5, high risk); Ki-67, proliferation index; TMTV, total metabolic tumor volume. Significance level, all *p* < 0.001.

**Figure 5:**
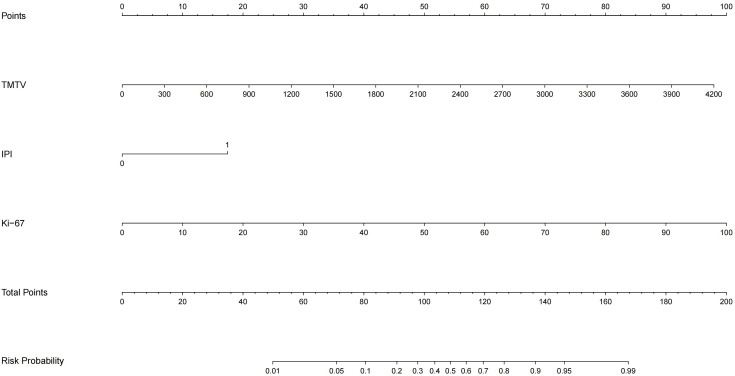
Nomogram of the TMTV+IPI+Ki-67 prediction model. TMTV, total metabolic tumor volume; IPI, International Prognostic Index (0–2, low risk; 3–5, high risk); Ki-67, proliferation index. The weight percentages of each variable are as follows, TMTV, 41.6%, IPI, 15.8%, Ki-67, 42.6%.

**Figure 6:**
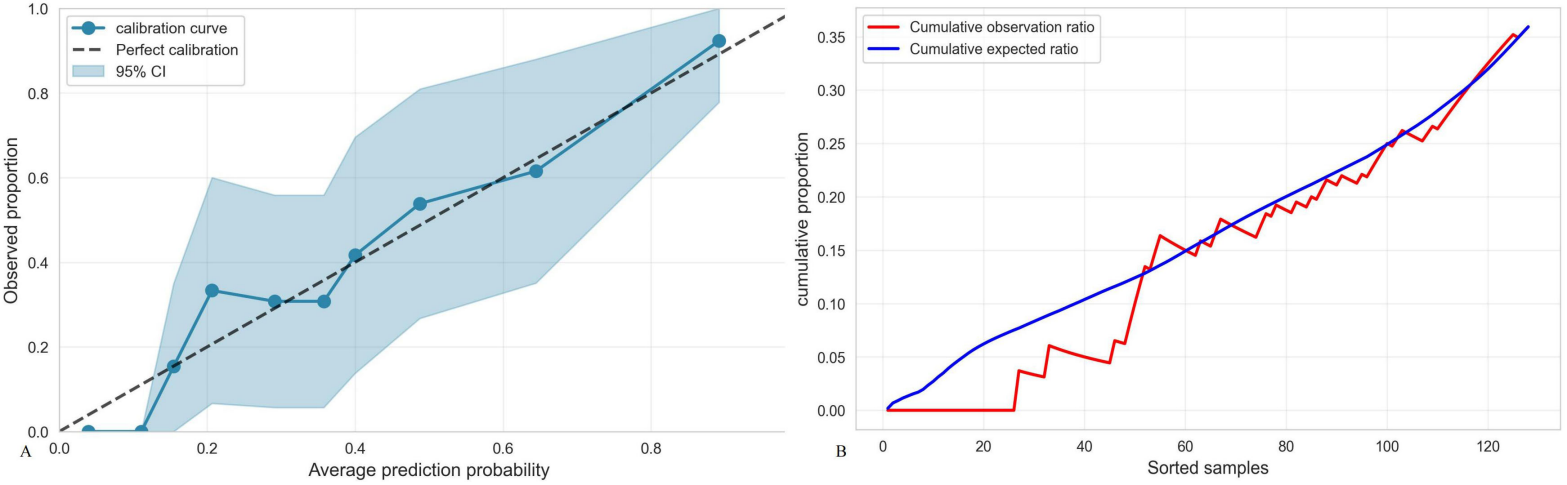
**(A)** Calibration curve of the TMTV+IPI+Ki-67 model. Brier points, 0.1585, Hosmer–Lemeshow test, 6.241, *p* = 0.620, Calibration slope, 0.142, Calibration intercept, 0.463, Calibration evaluation, good. **(B)** Accumulated calibration curve.

**Figure 7:**
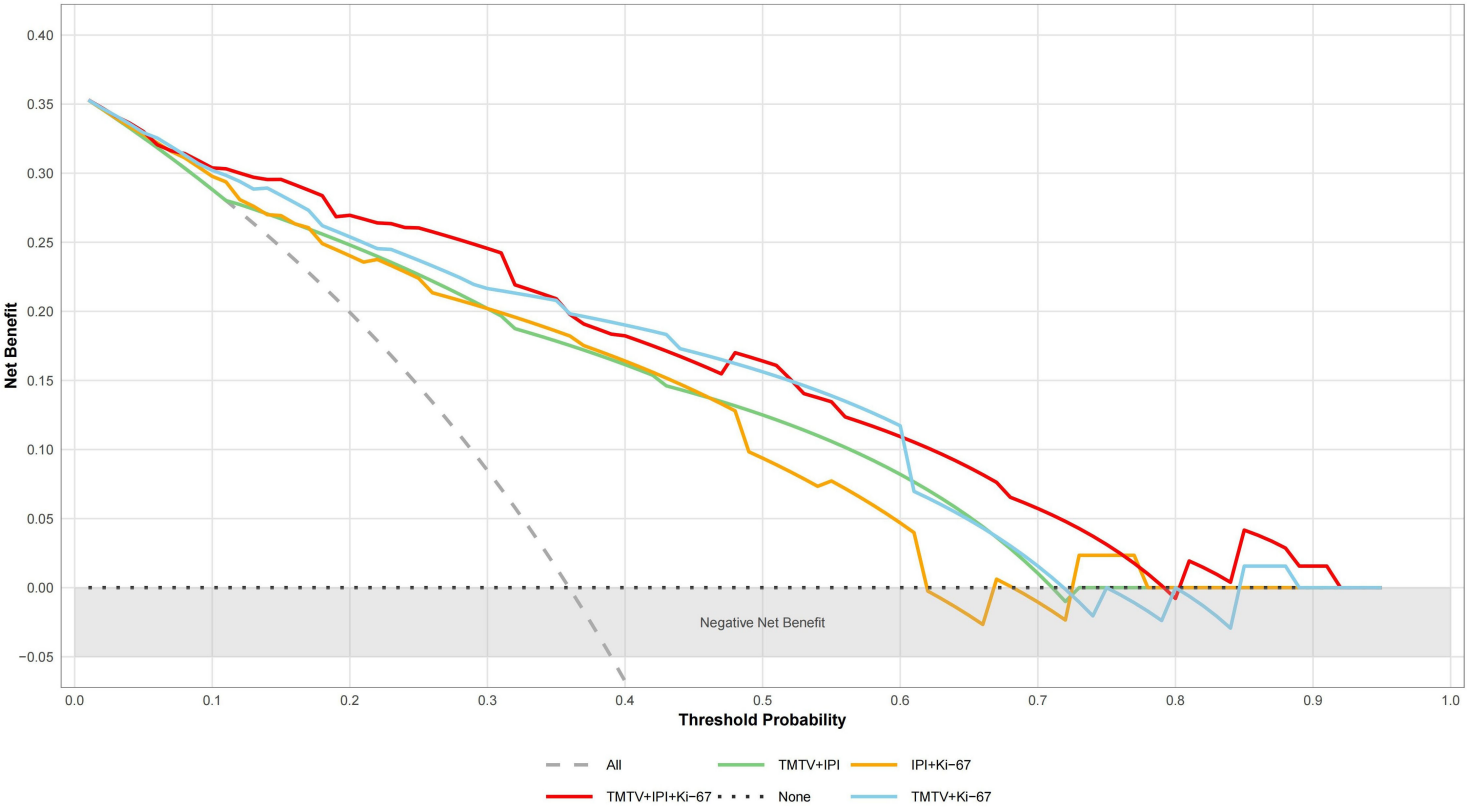
Analysis of decision curves of different DEL prediction models. “All” represents the assumption that treat all; “None” indicates the assumption that treat none. TMTV, total metabolic tumor volume; IPI, International Prognostic Index (0–2, low risk; 3–5, high risk); Ki-67, proliferation index.

TMTV, IPI, and Ki-67 were incorporated to develop a combined predictive model (TMTV+IPI+Ki-67). The model demonstrated significantly superior discriminative ability compared to all dual-parameter combinations (AUC, 0.867 vs. 0.790–0.844; all *p* < 0.05; **Figure 4**). Notably, TMTV emerged as the primary performance driver, dual-parameter models containing TMTV (TMTV+IPI, AUC = 0.798; TMTV+Ki-67, AUC = 0.844) outperformed the model without TMTV (IPI+Ki-67, AUC = 0.797). Visualization via nomogram (**Figure 5**) quantified predictor contributions, assigning relative weights of 41.6% (TMTV) and 42.6% (Ki-67), establishing both as core determinants of the model’s predictive function. Calibration validation revealed that the conventional calibration curve (**Figure 6A**) demonstrated good agreement within the intermediate-risk range (0.3–0.7; mean absolute error < 5%), but minor systemic miscalibration across the distribution (slope = 0.142, intercept = 0.463). The cumulative calibration curve (**Figure 6B**) indicated a maximum deviation <5% across the risk spectrum, with the Hosmer–Lemeshow test (*χ*² = 6.241, *p* = 0.620) confirming adequate overall calibration. This was further supported by a relatively low prediction error (Brier score = 0.158). DCA (**Figure 7**) further established the model’s clinical utility, yielding significantly higher net benefit (ΔNB = 3.4%–11.5%) across clinically relevant thresholds compared to alternative models.

## Discussion

Currently, lines of evidence indicate that the unique c-Myc/Bcl-2 co-expression signature in DEL synergistically activates the PI3K/AKT/mTOR pathway to drive metabolic reprogramming toward glycolysis. Mechanistically, c-Myc enhances glycolytic flux through upregulation of glucose transporter proteins (GLUTs), while Bcl-2 sustains cell survival by suppressing mitochondrial apoptosis, both collectively maintaining a hypermetabolic state (25–30). Additionally, the Myc-driven Warburg effect leads to lactate accumulation, inducing the formation of an immunosuppressive microenvironment. This is accompanied by abnormal elevation of serum LDH/β2-MG levels, which is associated with therapy resistance (25). This metabolic phenotype is closely correlated with PET/CT metabolic parameters (SUVmax/TMTV/TLG), a high SUVmax indicates increased glucose uptake capacity, while enlarged TMTV/TLG represents the cumulative tumor burden and potential for aggressive dissemination. This study further revealed a strong correlation between TMTV/TLG and LDH/β2-MG in the DEL subgroup. This finding, at the metabolic imaging level, unveils the interplay between metabolic burden and tumor biological activity, providing biological validation for the relevant imaging biomarkers.

Our clinical feature analysis confirmed that DEL patients exhibit a more aggressive biological behavior. Compared to the non-DEL group, DEL patients showed a significantly higher incidence of B symptoms, a greater proportion with IPI ≥ 3, a higher percentage in advanced Ann Arbor stage (III–IV), and a significantly elevated proportion of the non-GCB subtype—consistent with previous studies (5, 6). DEL patients simultaneously exhibited higher levels of Ki-67, LDH, and β2-MG, along with significantly increased SUVmax, TMTV, and TLG. We observed significant positive correlations between metabolic parameters (SUVmax/TMTV/TLG) and IPI, Ann Arbor stage, β2-MG, and LDH, validating that DEL embodies the biological essence of both high metabolic burden and strong proliferative activity.

Ki-67, a nuclear protein involved in cell proliferation regulation, is widely utilized as a biomarker for assessing proliferative activity in lymphoma. Counterintuitively, this study observed that Ki-67 in DEL patients demonstrated a statistically significant association only with extranodal site, while showing no statistically significant correlation with the IPI or metabolic parameters (SUVmax/TMTV/TLG). However, multivariate logistic regression analysis definitively confirmed Ki-67 as an independent predictor of DEL. This phenomenon may be partially attributable to limited sample size, but the more critical underlying mechanism likely involves c-Myc/Bcl-2-driven metabolic reprogramming potentially operating partially independent of proliferation regulation pathways—meaning the tumor cells’ survival advantage may be preferentially achieved through energy metabolism pathways, where proliferative activity in specific DEL subtypes could be masked by metabolic dysregulation. Precisely because of the biological distinctiveness and complementarity between the proliferative potential reflected by Ki-67, the metabolic burden quantified by TMTV, and the clinical status assessed by IPI, these three factors contribute unique incremental value in the combined predictive model. The controversy regarding Ki-67’s correlations in lymphoma research (31–33) further highlights the limitations of traditional single indicators, strongly underscoring the necessity for multimodal predictive models.

While the IPI remains a widely used risk stratification tool for lymphoma patients (34), its reliance on static clinical parameters, such as age and disease stage, fails to capture the dynamic metabolic characteristics of DEL. Ruppert et al. (35) have proposed integrating additional molecular markers to refine risk stratification. In contrast, TMTV quantifies metabolically active tumor burden, enabling longitudinal tracking of disease progression and systemic tumor load with temporal resolution.

This study identified TMTV > 510.22 cm³ as an independent predictor of DEL. While prior studies established MTV thresholds for survival prognostication (17, 18), our work extends TMTV’s utility to DEL phenotype prediction—a distinct clinical context requiring phenotype-specific stratification. TMTV’s role as a volumetric biomarker of tumor burden conceptually aligns with its established value in DLBCL aggressiveness, operationalized here for molecular subtyping. Specifically, Eertink et al. (17) and Ceriani et al. (36) demonstrated that integration of TMTV significantly enhanced the discriminatory capacity of risk stratification models. These findings collectively support the critical role of baseline PET/CT metabolic parameters, particularly TMTV, in compensating for the deficiencies of traditional clinical indicators.

This study has the following limitations. First, as a retrospective single-center study, it may be inherently susceptible to selection bias. For example, the proportion of DEL patients in this study cohort was 35.9% (46/128), which is slightly elevated compared to the commonly reported range of 20%–30% in newly diagnosed DLBCL cases (2, 3). This discrepancy may stem from patient selection criteria or potential geographic/ethnic factors. Importantly, despite this proportional difference, the DEL group exhibited highly aggressive clinicopathological features and enhanced metabolic activity, findings that closely align with previous reports on DEL (4–6). This consistency supports the robustness of DEL subgroup identification in this cohort. Second, the limited sample size (*n* = 128) and reliance solely on internal evaluation without external independent cohort validation or rigorous internal cross-validation may compromise the assessment of model generalizability and reduce the statistical power of subgroup analyses. Finally, this study utilized ^18^F-FDG, the current standard for DLBCL metabolic imaging. Future research could explore novel tracers like ^18^F-FAPI for specific biological niches—particularly fibroblast-rich microenvironments or FDG-avid lesions where dual-tracer imaging may elucidate stromal contributions to treatment resistance. Such mechanistic studies would complement (rather than replace) FDG-based diagnostic paradigms (37). Future research will require prospective, multicenter, large-sample cohort studies, coupled with independent external validation, to more accurately define the epidemiological characteristics and predictive factors of DEL, and to validate the robustness and clinical applicability of this combined predictive model.

The combined predictive model developed in this study significantly enhanced the early identification of DEL in internal validation, outperforming conventional clinical stratification approaches. This multidimensional model not only provides a novel strategy for the non-invasive screening of treatment-naïve patients most likely to benefit from targeted intensified therapy, but also underscores the superior clinical utility of TMTV compared to conventional SUVmax. While calibration deviations occur and clinical applicability requires further prospective validation, the model exhibits significant potential for precise identification of high-risk DEL patients during therapeutic decision-making, thereby optimizing individualized treatment strategies. In summary, this model is readily implementable in hospitals with PET/CT capabilities and standard DLBCL diagnostic workflows. Multi-center validation (planned) will further establish generalizability.

## Data Availability

The original contributions presented in the study are included in the article/supplementary material. Further inquiries can be directed to the corresponding authors.
